# One-year results of visual response following intravitreal novel anti-VEGF injection for diabetic macular edema in a Latino population

**DOI:** 10.1186/s40942-025-00719-9

**Published:** 2025-08-01

**Authors:** Guillermo Salcedo-Villanueva, Gisela Garcia-Sánchez, Claudia Palacio-Pastrana, Gerardo Gascón-Guzmán, Aureliano Moreno-Andrade, Oscar Olvera-Montaño, Patricia Muñoz-Villegas

**Affiliations:** 1 Asociación para Evitar la Ceguera en México, I.A.P., Mexico City, Mexico; 2 Regional Medical Affairs Department, Laboratorios Sophia, S.A. de C.V., Paseo del Norte 5255, Guadalajara Technology Park, Zapopan, Jalisco 45010 Mexico; 3 SalaUno Salud, S.A.P.I. de C.V., Mexico City, Mexico; 4 Consultorio de Medicina Especializada, Nuevo Laredo, Tamaulipas, Mexico; 5 Retina Center, Tijuana, Baja California Mexico

**Keywords:** Central macular thickness, Diabetes mellitus, Diabetic macular edema, Visual acuity, anti-VEGF

## Abstract

**Background:**

Diabetic macular edema (DME) is a leading cause of vision impairment. This study evaluated the effects of multiple anti-VEGF intravitreal injections, including a novel anti-VEGF PRO-169, on best-corrected visual acuity (BCVA) and central macular thickness (CMT) in Latino patients with DME.

**Methods:**

This is a multicenter, drug-agnostic interim analysis. Patients were randomized 1:1 to receive monthly injections for four months after a pro re nata intravitreal injection of either PRO-169 or ranibizumab over a one-year period.

**Results:**

A total of 278 eyes with DME were analyzed. The average age of the participants was 62.1 ± 7.4 years, with diabetes diagnosed at an average of 16.7 ± 8.4 years and DME detected 1.2 ± 1.6 years later. By week 48, patients with an initial BCVA letter score of < 69 experienced a gain of 13.1 ± 10.4 letters, with an injection frequency of 34.5 ± 6.9 days per injection. The group showed a reduction in CMT of -127 ± 153 μm, compared to a -82.2 ± 82.1 μm reduction in those with an initial BCVA between 69 and 78 letters (*p* = 0.016). Additionally, 49% of patients with an initial score < 69 letters improved their visual acuity to 20/40 or better, and 41.5% gained 15 or more letters.

**Conclusions:**

This interim analysis indicates the potential effectiveness of the anti-VEGF agents PRO-169 and ranibizumab, especially for patients with initial visual acuity < 69 letters. The final analysis will be essential for verifying the efficacy and safety of PRO-169. This study provides solid evidence to support ophthalmologists treating Latino patients with DME and likely improves patient care.

**Trial registration:**

NCT05217680 (clinicaltrials.gov).

**Supplementary Information:**

The online version contains supplementary material available at 10.1186/s40942-025-00719-9.

## Background

Diabetic macular edema (DME) is a visually threatening condition that affects around 4–7% of patients with diabetic retinopathy (DR) worldwide and remains the leading cause of blindness in the working-age population [1–3]. Latinos with primarily Mexican, Dominican, and Puerto Rican ancestry exhibit a high prevalence of DR and DME [4–8]. DME can develop in any stage of DR [6]; however, its prevalence is positively correlated with the duration of diabetes and the severity of DR. Other systemic and ocular factors such as hypertension, higher glycated hemoglobin (HbA1c) levels, hyperlipidemia, and previous cataract surgery have been established as contributing determinants in the condition [9–13]. Therefore DME is a multifactorial disorder derived from the activation of several signaling pathways leading to the formation of reactive oxygen species, overexpression of inflammatory markers and upregulation of vascular endothelial growth factor (VEGF); consequently, pathological alterations in the retinal neovascular units and disruption of the blood-retinal barrier, collectively contribute to retinal capillary damage, vascular hyperpermeability, and increased activity of inflammatory processes that promote extravasation and accumulation of fluid and serous macromolecules in the intra- and extracellular space. These changes induce structural and functional abnormalities in the macular area, leading to central vision loss [14–20]. As DME treatment is crucial for preventing severe vision loss, intravitreal anti-VEGF injections are considered the first-line therapy [13, 16, 17, 21, 22]. These drugs inhibit proangiogenic factors’ activity and facilitate the preservation of an edema-free environment around the macula, favoring normalization of the neurovascular units and neuroprotection [13, 17]. Clinical trials have consistently demonstrated the considerable benefits of these agents for patients with DME [23–28]. However, evidence also highlights the high cost of these medications and their significant economic impact on patients in real-world settings [29–32]. For this reason, we aimed to evaluate a new anti-VEGF drug alternative that could reduce treatment costs while maintaining efficacy in managing DME. This study randomized patients to receive intravitreal injections of either PRO-169 or ranibizumab (IVR), two anti-VEGF agents. PRO-169 is a recombinant, humanized monoclonal antibody that targets VEGF-A. It shares its sequence with commercially available bevacizumab, but it was specifically developed for intravitreal use. PRO-169 has demonstrated consistency with historical bevacizumab data in terms of quality, heterogeneity profile, purity, molecular structure, and binding affinity. Non-clinical studies have shown that intravitreal PRO-169 has a similar profile to the bevacizumab innovator (Avastin^®^, Roche, Basel, CH) in terms of tolerability, pharmacokinetics, and its ability to inhibit damage from laser-induced choroidal neovascularization. Additionally, it is presented in a pre-filled syringe to simplify administration and reduce the risk of endophthalmitis [33–36]. Ranibizumab is a Fab fragment of bevacizumab developed for intravitreal injections. It is approved for wet age-related macular degeneration, retinal vein occlusion, DME, DR, and myopic choroidal neovascularization [25, 37–39]. It is essential to select the most suitable treatment option for DME based on clinical evidence and considering individual patients’ general health, ocular condition, motivations, and compliance with treatment in real-world clinical practice [40]. We hypothesized that patients with DME and mild visual acuity loss would exhibit different responses to anti-VEGF treatments, considering factors such as longer diabetes duration, age, sex, and other common variables.

The current study aimed to investigate changes in best-corrected visual acuity (BCVA) and central macular thickness (CMT) in Latino patients with DME who received multiple intravitreal injections of anti-VEGF (either PRO-169 or ranibizumab) over one year in a multicenter setting.

## Methods

A one-year, drug-agnostic, interim, observational, and descriptive analysis was performed using a multicenter Phase III clinical trial (ClinicalTrials.gov Identifier: NCT05217680, with an estimated completion date of late 2025) involving 20 centers in Mexico (see the Acknowledgements section for details). The primary study included patients who met the inclusion criteria and signed an informed consent form for all the procedures. The protocol adheres to the principles outlined in the Declaration of Helsinki, ICH guidelines, and current local regulations. The cohort was recruited between July 2021 and August 2023.

The inclusion criteria mandated that participants have a diagnosis of DME with clinical evidence of central macular thickening or DME present on spectral domain optical coherence tomography (OCT, criterion of CMT [measured in the 1-mm diameter central subfield] > 300 μm for men and > 290 μm for women), older than 18 years, male or female, with HbA1c value < 9.5%. The ocular inclusion criteria included a corrected visual acuity of Early Treatment Diabetic Retinopathy Study (ETDRS) < 78 letters (equivalent to approximately 20/32 or worse) and > 24 letters (approximately 20/230 or better). The exclusion criteria were chronic kidney disease in renal failure, requiring glycemic control with insulin, poorly controlled blood pressure, history of myocardial infarction or another cardiovascular event, and non-diabetic macular edema, intraocular pressure (IOP) > 21mmHg, lens opacities, evidence of macular traction and hyaloid thickening on OCT. See the Supporting Material section (Table S1) for more information about inclusion and exclusion criteria.

In this study, one eye per patient was randomly allocated in a 1:1 ratio to receive monthly intravitreal injections of PRO-169 or IVR for four months. Subsequently, a *pro re nata* treatment regimen was implemented over the course of one year, guided by individual patient response. Retreatment decisions were based on changes in BCVA of > 5 or < 5 ETDRS letters and/or CMT variations of > 10% or < 10% from the previous assessment. Patients who exhibited fluctuations below these thresholds were classified as “stable” and did not receive an intravitreal injection at that visit. The sponsor developed the randomization specifications, and a third party programmed and held the algorithm. All investigators and other team members involved in the study were blinded to treatment assignment throughout the study. In each center, the unblinded team was responsible for dispensing the study drug to the ophthalmologist, who remained unblinded and performed all injections. Each patient was assessed monthly by the blinded team through a complete ophthalmologic examination, including a BCVA test according to ETDRS guidelines, biomicroscopy for detailed assessment of ocular structures and media transparency, and indirect ophthalmoscopy for detailed retinal examination. Subsequently, an OCT was conducted to assess the CMT [Heidelberg Spectralis OCT (Heidelberg, Germany), Zeiss Cirrus HD-OCT 5000–6000 (Zeiss Meditec, Dublin, CA), and 3-D OCT-1000 (Topcon, Tokyo, Japan)]. The patient’s age, sex, weight, body mass index, waist circumference, and a brief questionnaire were utilized to obtain information about their medical history, including systemic hypertension, diabetes mellitus, and DME diagnosis. At each center, all measurements were taken under the same conditions and with the same instruments for every patient.

The study aimed to compare patients’ responses regardless of their initial BCVA. When evaluating the reactions to anti-VEGF agents in patients with varying levels of visual impairment, several factors must be considered, including the number of injections, baseline CMT, short-term response, treatment response, and functional response. Based on these factors, the primary outcomes assessed at 48 weeks included the mean change from baseline in BCVA and from baseline in CMT over time. Secondary endpoints included the mean number of injections over time, the proportion of eyes that gained at least 10 letters from baseline, the proportion of eyes that gained 15 or more letters from baseline, and the proportion of patients achieving a BCVA Snellen equivalent of 20/40 vision or better.

Patients who discontinued the clinical trial with less than 36 weeks of follow-up were excluded from the analysis. For more information about those patients, please refer to Table S2.

### Statistical analysis

This drug-agnostic interim analysis was conducted under blinded conditions, and the coding for the assignments was not accessible to the investigators, specific sponsor members, or statisticians involved in the analysis. The data was managed using R statistical software (The R Foundation for Statistical Computing; http://www.R-project.org). Missing values were imputed using the last observation carried forward method. Descriptive statistics, such as means, frequencies, and standard deviations (SD), were used to organize, summarize, and present all the data unless otherwise specified. The data were categorized into two groups based on their initial BCVA letter score: <69 and between 69 and 78. This classification aligns with the findings of Protocol T [41]. Differences were assessed using the student’s t-test for continuous data. Ordinal variables were analyzed using p x q contingency tables, and the differences were managed using Pearson’s Chi-square test. All statistical analyses performed in this study had a threshold of *p* < 0.05.

## Results

### Characteristics of the participants

Three hundred fifty-three patients were enrolled and randomized between July 2021 and August 2023. By week 48, 78.7% of patients had completed the cohort of a one-year study period. The analysis included 278 eyes belonging to 278 subjects (145 men and 133 women) with type I or II diabetes and diagnosed with DME (intent-to-treat population). The overall baseline clinical characteristics and demographic data of patients are detailed in Table 1. The mean age was 62.1 ± 7.4 years; 71.9% (200 out of 278) of the patients had baseline BCVA letter scores of < 69 letters (equivalent to 20/50 or worse). Additionally, 45% (125 out of 278) of them had controlled HbA1c (≤ 7%). The mean value of HbA1c was 7.15 ± 0.85%. On average, the time since the diagnosis of diabetes mellitus was 16.7 ± 8.4 years, and the duration with DME was 1.2 ± 1.6 years. The average CMT was 430 ± 114 μm.

**Table 1:** Overall baseline clinical characteristics and demographic data of patients (*n* = 278)

Characteristic		< 69 letters	> 69 letters	*p*-value
Number of patients (eyes)		200	78	…
Age, years ± SD		62.5 ± 7.0	61.1 ± 8.1	0.143^1^
Sex, M/F (%)		101 (50.5) / 99 (49.5)	44 (56.4) / 34 (56.4)	0.375^2^
BMI, kg/m^2^ ± SD		27.5 ± 4.2	27.6 ± 4.8	0.954^1^
Waist circumference, cm ± SD		92.0 ± 14.8	94.2 ± 14.2	0.267^1^
Systemic Hypertension, n (%)		128 (64)	48 (61.5)	0.702^2^
Other comorbidities, n (%)		190 (95)	77 (98.7)	0.153^2^
Glycosylated hemoglobin, g/dL %		7.2 ± 0.85	7.0 ± 0.84	0.159^1^
≤ 7%, n (%)		84 (42)	41 (52.6)	0.112^2^
> 7%, n (%)		116 (58)	37 (47.4)	
Diagnosis of DM, years ± SD		16.7 ± 8.6	16.6 ± 8.0	0.934^1^
Diagnosis of DME, years ± SD		1.1 ± 1.5	1.4 ± 1.8	0.176^1^
Central macular thickness, µm ± SD		450 ± 121	380 ± 71.9	< 0.001^1^
BCVA-ETDRS letters ± SD		52.8 ± 11.6	73.1 ± 2.4	< 0.001^1^
BCVA-LogMAR ± SD		0.64 ± 0.24	0.24 ± 0.05	< 0.001^1^
Snellen BCVA, n (%)	20/40 or better	17 (8.5)	78 (100)	< 0.001^2^
	20/50 to 20/70	65 (32.5)	…	
	20/80 to 20/200	112 (56)	…	
	20/200 or worse	6 (3)	…	
IOP, mmHg ± SD		14.6 ± 2.1	14.4 ± 2.2	0.465^1^

**Notes**: ^1^t-test. Chi-square test, ^2^Chi-square test

**Abbreviations**: BCVA, best corrected visual acuity; BMI, Body mass index; DM, diabetes mellitus; DME, diabetic macular edema; IOP, intraocular pressure; F, female; M, male; SD, standard deviation

### Mean 1-year change in BCVA and mean of anti-VEGF injections

**Fig. 1:**
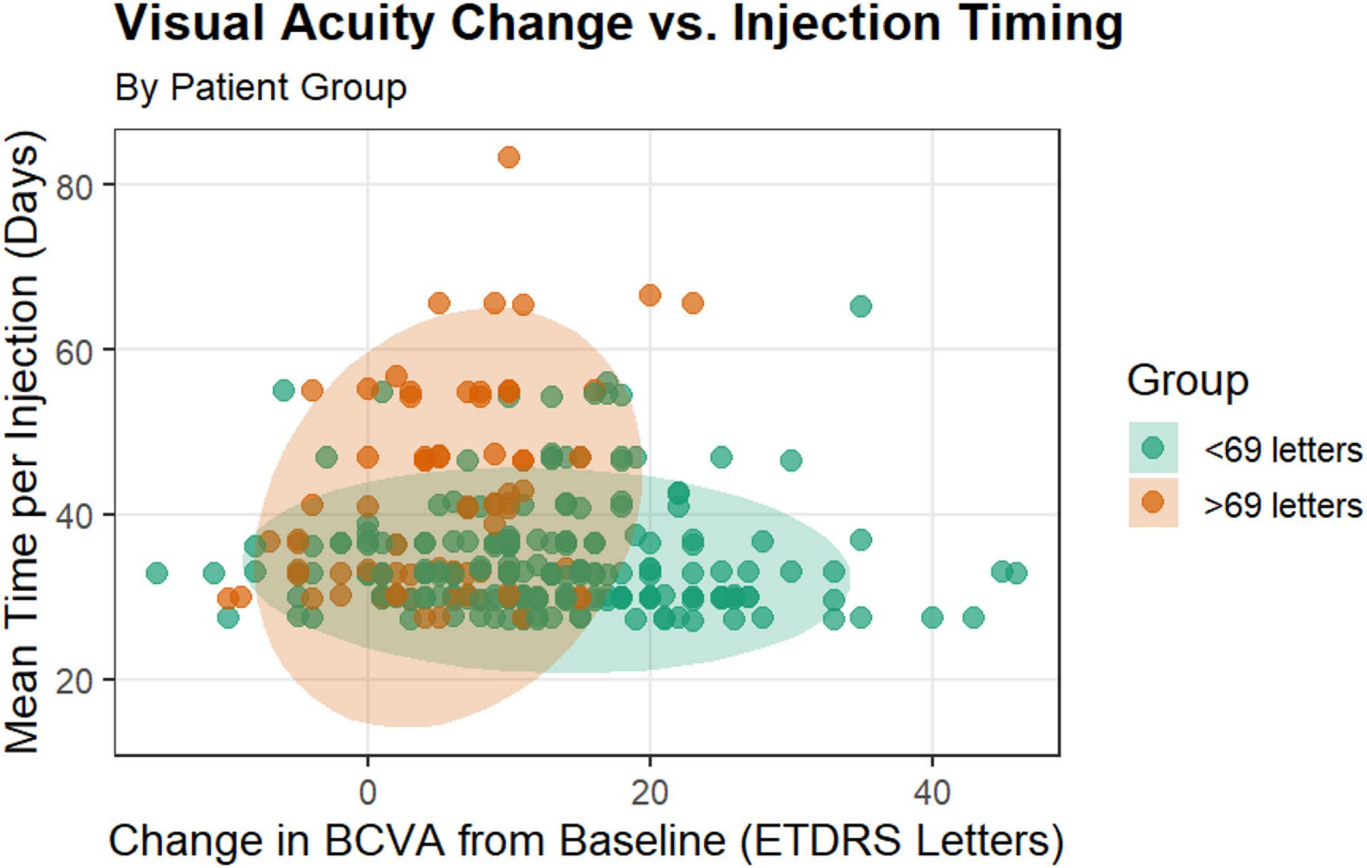
The relationship between best-corrected visual acuity (BCVA), measured in ETDRS letters, changes after one year of intravitreal injections for patients with an initial BCVA score of < 69 than those with an initial BCVA of 69 to 78. A statistically significant difference was observed between the two groups, with a p-value of less than 0.001. The scatter plot illustrates the potential variances of the adjusted x- and y-coordinates at a 95% confidence level

By week 48, the group with a baseline BCVA of < 69 letters had an average score of 65.8 ± 12.4 letters. In comparison, patients with baseline BCVA between 69 and 78 letters had an average score of 78.7 ± 6.4 letters (*p* < 0.001). This change indicates an average gain of 13.1 ± 10.4 letters for patients with an initial score of < 69 and an increase of 5.6 ± 6.4 for those with initial BCVA scores between 69 and 78 (*p* < 0.001). As shown in Fig. 1, patients with an initial score of < 69 required more frequent intravitreal injections to improve their BCVA and maintain disease control. In contrast, patients with initial scores of 69 and 78 required fewer injections to manage their condition. The average number of anti-VEGF injections per patient was 9.8 ± 1.6 for those with an initial score of < 69, compared to 8.6 ± 2.1 for those between 69 and 78 (*p* < 0.001); see Fig. S1. This translates to an average injection frequency of 34.5 ± 6.9 days and 41.0 ± 12.1 days, respectively (*p* < 0.001); see Table 2 for further details. Additionally, the percentage of patients with well-controlled HbA1c levels in the group with baseline BCVA < 69 letters remained stable over the 48 weeks. However, for patients with baseline BCVA between 69 and 78 letters, the percentage of those with HbA1c levels above 7% increased to 13% by week 48. The average HbA1c for uncontrolled patients was 8.7 ± 1.6% in the group with a baseline BCVA of less than 69 letters, compared to 8.5 ± 1.5% for patients with a baseline BCVA between 69 and 78 letters. There were no significant differences between the two groups at any time regarding changes in BCVA, regardless of the patients’ HbA1c levels; see Fig. S2 for more information.

**Table 2:** Mean 1-year changes, stratified by initial VA score

Characteristics		< 69 letters	> 69 letters	*p*-value
Mean of anti-vascular endothelial growth factor Injection per patient ± SD		9.8 ± 1.6	8.6 ± 2.1	< 0.001^1^
Anti-vascular endothelial growth factor injection frequency, days ± SD		34.5 ± 6.9	41.0 ± 12.1	< 0.001^1^
Gain of ≥ 10 letters, n (%)		48 (24)	19 (21.4)	< 0.001^2^
Gain of ≥ 15 letters, n (%)		83 (41.5)	6 (7.8)	
BCVA-ETDRS letters ± SD		65.8 ± 12.4	78.7 ± 6.4	< 0.001^1^
Changes in BCVA, n (%)	20/40 or better	98 (49)	75 (96.2)	< 0.001^2^
	20/50 to 20/70	61 (30.5)	3 (3.8)	
	20/80 to 20/200	41 (20.5)	…	
Glycosylated hemoglobin, g/dL %		7.6 ± 1.8	7.6 ± 1.7	0.919^1^
≤ 7%, n (%)		90 (45)	31 (39.7)	0.427^2^
> 7%, n (%)		110 (55)	47 (60.3)	
Mean change of IOP, mmHg ± SD		-0.18 ± 1.8	-0.19 ± 1.7	0.975^1^
Mean change of CMT, µm ± SD		-127 ± 153	-82.2 ± 82.1	0.016^1^

**Notes**: ^1^t-test. Chi-square test, ^2^Chi-square test

**Abbreviations**: BCVA, best corrected visual acuity; CMT, Central macular thickness; IOP, intraocular pressure; SD, standard deviation

### Mean 1-year change in CMT and BCVA

**Fig. 2:**
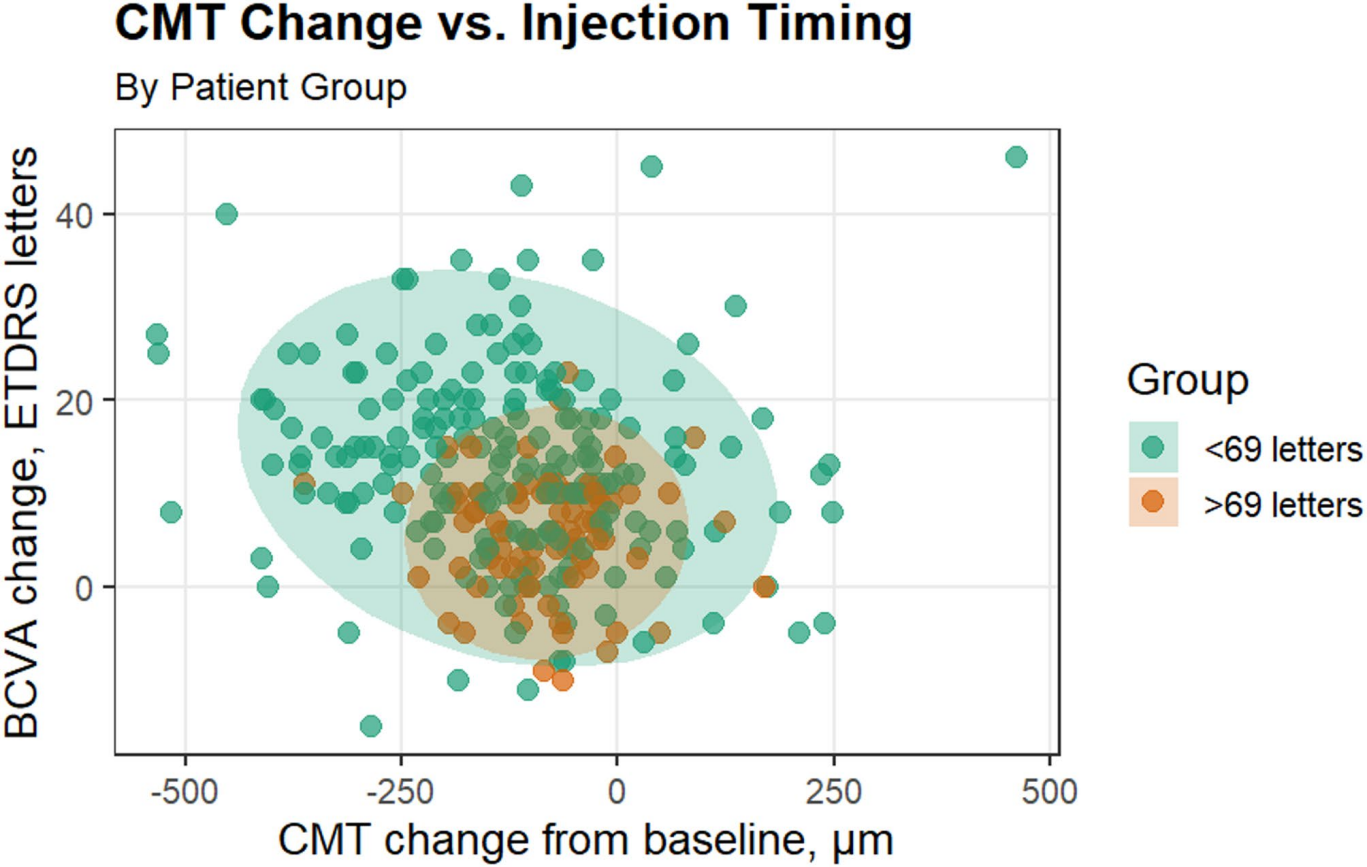
The relationship between central macular thickness (CMT) and best-corrected visual acuity (BCVA) changes one year after intravitreal injections. This variation is particularly significant among patients with an initial BCVA letter score of < 69 compared to those with an initial BCVA of 69 to 78 letters. A statistically significant difference exists between these two groups, with a *p*-value of less than 0.001. The accompanying scatter plot illustrates the potential variations in the adjusted x- and y-coordinates at a 95% confidence level

By week 48, there was a decrease in the average CMT for both patient groups. The mean CMT values were 323 ± 122 μm for patients with an initial BCVA score of < 69 letters, compared to 297 ± 69.5 μm for patients with initial BCVA scores between 69 and 78 letters (*p* = 0.083). This change indicates an average reduction of -127 ± 153 μm for patients with initial scores under 69 letters and − 82.2 ± 82.1 μm for those scoring between 69 and 78 letters (*p* = 0.016). Figure 2 shows the relationship between CMT change and BCVA change after one year of anti-VEGF injections. The change in CMT was more significant in patients with an initial score of 69 or less, as shown in Table 2. Patients with baseline BCVA < 69 letters required more frequent injections to decrease their CMT and maintain disease control; conversely, patients with initial BCVA scores between 69 and 78 letters required fewer frequent injections to maintain disease control, as illustrated in Fig. S3. The study compared the prevalence of systemic hypertension (64% vs. 61.5%, *p* = 0.702) and the duration after diagnosis of diabetes mellitus (16.7 ± 8.6 years vs. 16.6 ± 8.0 years, *p* = 0.934) to assess their impact on the change in CMT. The results showed that patients with high blood pressure (HBP) tended to experience an increase in CMT when they had had diabetes mellitus for a longer period. Conversely, patients without this comorbidity experienced a significant decrease in CMT over time, as illustrated in Fig. S4.

### One-year BCVA gain ETDRS letters

**Fig. 3:**
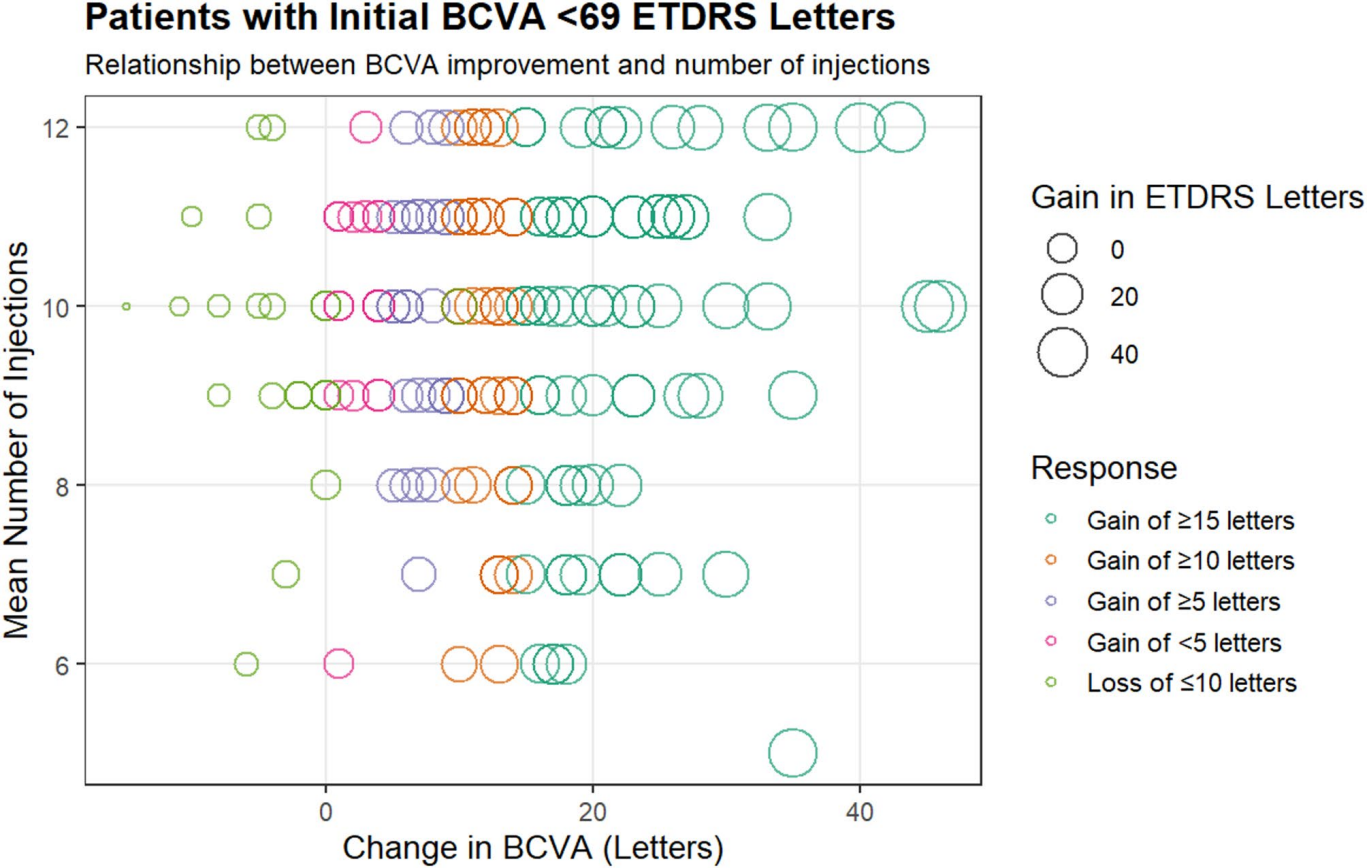
Best-corrected visual acuity (BCVA) changes were observed for patients with an initial BCVA letter score of < 69. The color of the bubbles indicates the response classification, while the size of each bubble represents the number of ETDRS letters gained or lost compared to the baseline

**Fig. 4:**
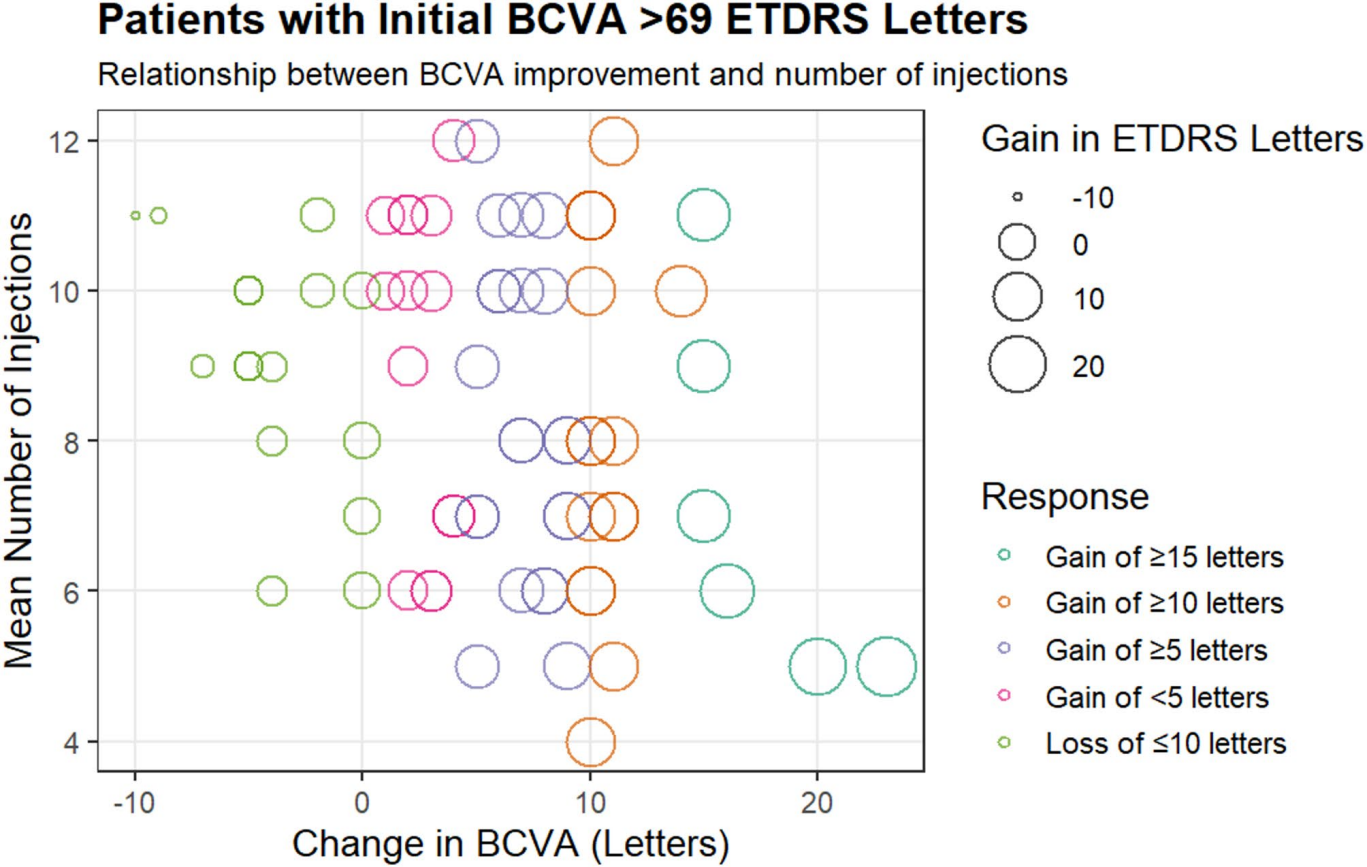
Best-corrected visual acuity (BCVA) changes were observed for patients with an initial BCVA of 69 to 78 letters. The color of the bubbles indicates the response classification, while the size of each bubble represents the number of ETDRS letters gained or lost compared to the baseline

Patients with initial scores under 69 letters experienced a significant increase in ETDRS letters from baseline by week 48 (*p* < 0.001). Notably, 24% of these patients gained at least 10 letters, comparable to the 21.4% with initial BCVA scores between 69 and 78. Among those with an initial BCVA score of < 69 letters, 41.5% gained 15 or more letters (see Fig. 3). In contrast, only 7.7% of patients with an initial BCVA score between 69 and 78 achieved 15 or more (*p* < 0.001). Patients with initial scores > 69 letters, averaging 73.1 ± 2.4 ETDRS letters at the start, were expected to have fewer letters to gain, as shown in Fig. 4. Furthermore, 11% of patients with baseline BCVA < 69 letters lost 10 or more letters, while 20.5% of those with a baseline BCVA between 69 and 78 letters also experienced a loss of 10 or more letters (*p* < 0.001). A more significant percentage of patients with initial scores < 69 letters achieved a Snellen BCVA of 20/40 or better at 48 weeks, rising to 49% from a baseline of 8.5%. By week 48, only 20.5% of patients in this group had a Snellen BCVA between 20/80 and 20/200 (see Table 2).

### Safety

Both groups of patients exhibited similar changes in IOP over time, regardless of the injection frequency (refer to Fig. S5). The mean change in IOP for patients with an initial BCVA score of < 69 letters was − 0.18 ± 1.8 mmHg, with an average of 9.8 ± 1.6 injections per patient. In contrast, patients with initial BCVA scores between 69 and 78 letters had a mean change of -0.19 ± 1.7 mmHg, receiving an average of 8.6 ± 2.1 injections each. The p-value for this comparison was 0.975 (see Table 2). Furthermore, the findings revealed that when comparing the impact of systemic hypertension prevalence and the duration following a diabetes mellitus diagnosis on changes in BCVA, there was no significant difference in BCVA changes between patients with systemic hypertension and those without (see Fig. S6).

In the intent-to-treat analysis involving 278 patients, 37.1% experienced at least one AE (103/278). Of these AEs, 18.9% were classified as mild, 68.2% as moderate, and 12.9% as severe. Six patients discontinued their participation between weeks 40 and 48 due to AEs. There were a total of 23 serious adverse events (20 systemic and three ocular), representing 13.5% of all adverse events. These included three fatalities—two caused by septic shock and one from an unknown cause. The ocular SAEs involved one case of diabetic neuropathy and two cases of endophthalmitis. Ocular events accounted for 30% of the total AEs, amounting to 51 incidents. The most reported AE was hypertensive emergency, occurring in 7.7% of patients, followed by dry eye disease, diabetic foot complications, and pharyngitis, each at 4.1% (see Table 3).

**Table 3:** Ocular and systemic adverse events of interest*

	Participants with one study eye (*n* = 103)
**Systemic Adverse Events (events occurring at least once)**	
Hypertensive emergency, n (%)	13 (10.9)
Diabetic foot, n (%)	7 (5.9)
Pharyngitis, n (%)	7 (5.9)
COVID-19, n (%)	5 (4.2)
Flu, n (%)	5 (4.2)
Death from any cause, n (%)	3 (2.5)
Any event, n (%)	79 (66.4)
Serious adverse event, n (%)	20 (16.8)
**Ocular Events (events occurring at least once)**	
Dry eye disease	7 (13.7)
Conjunctivitis	5 (9.8)
Subconjunctival hemorrhage	4 (7.8)
Vitreous hemorrhage	4 (7.8)
Elevation in intraocular pressure	3 (5.9)
Cataract surgery	3 (5.9)
Any ocular event	25 (49.1)
Serious adverse events	3 (5.9)

*Unless otherwise specified, adverse events were collected during study follow-up

## Discussion

Diabetes mellitus is a chronic disease associated with a greater risk of vascular complications, end-stage renal disease, DR, and lower extremity amputations. The Latino population, especially those with Mexican, Dominican, and Puerto Rican backgrounds, has a disproportionate burden of diabetes-related complications, attributable to both behavioral factors (such as a less active lifestyle) and genetic predispositions (including greater adiposity and a tendency toward insulin resistance) [7, 8]. Focusing specifically on DR, Latino patients have a higher prevalence, earlier disease onset, and baseline severity of this diabetes-related complication and its sequelae, including DME. The exact prevalence and treatment response of DME in the Latino population is uncertain, owing to the lack of recent research conducted in Latin America and the underrepresentation of this ethnic group in studies conducted in the US [42–44].

Previous studies have indicated a relationship between higher rates of cumulative DR progression to more advanced visual-threatening stages, including DME, as well as factors such as longer duration of diabetes, age, and male population [45, 46]. However, in our study, these three variables did not show statistical significance in patients with an initial BCVA score of < 69 letters compared to those who started with initial BCVA scores between 69 and 78.

Different patient responses occur at the onset of therapy, regardless of their baseline BCVA. To date, this method has not been explored for PRO-169, a monoclonal antibody targeting VEGF for intravitreal administration [33, 34]. Investigating the efficacy and safety of PRO-169 is essential, as it may offer an alternative that alleviates the social and economic burden on patients undergoing multiple drug administrations. This is of particular significance to the Latino population, which has a high incidence of DME and frequently delayed access to treatment [47, 48]. Tight control of glycemic levels and HBP has been proven to reduce the risk of DME and the progression of DR [49, 50]. However, once DME has developed, the evidence regarding the influence of metabolic control on its severity or response to anti-VEGF treatments remains uncertain. Studies by Peng and Tsai (2018), Macky and Mahgoub (2012), and Matsuda et al. (2014) suggest that higher levels of HbA1c (> 7%) are associated with lower macular thickness in eyes affected by DME [49–51]. Additionally, Bansal et al. (2015) conducted a *post hoc* analysis of the RIDE/RISE trials, considering patients’ baseline HbA1c levels and their progression over a 36-month follow-up period. Patients were stratified into subgroups based on whether their HbA1c values improved, remained stable, or worsened throughout the study duration. They reported overall visual and anatomic improvement in patients treated with monthly anti-VEGF injections, independent of their baseline HbA1c. There was no statistical difference among the three subgroups of patients with improved, stable, or worsened HbA1c throughout the study [52]. These reports align with our findings, as no significant disparity was observed between the HbA1c levels (≤ 7% and > 7%) and BCVA letter scores at any point during one year of anti-VEGF treatment.

Regarding HBP, even in the absence of clinical signs related to DR, patients with both diabetes mellitus and HBP have impaired macular microcirculation and microvasculature. Additionally, inadequate blood pressure control increases the likelihood of DR or DME progression by 91% and 40%, respectively [53–55]. For every 10-mmHg augmentation in systolic blood pressure, the risk of DME increased by 6% (HR 1.06, 95% CI, 1.02–1.09). Moreover, elevated systolic blood pressure has been significantly associated with the need for anti-VEGF treatment in patients with diabetes [56, 57]. Our study assessed the correlation between diabetes mellitus duration in patients with or without hypertension (16.7 ± 8.6 years vs. 16.6 ± 7.9 years, respectively; *p* = 0.934) and the CMT. Additionally, 63.3% of the patients had been diagnosed with systemic hypertension, all of which were identified as risk factors before the study [12, 58]. The results exhibited a significant decrease in CMT over time in patients without hypertension, which suggests that this comorbidity could negatively impact treatment response in patients with DME.

BCVA and OCT morphological analysis are the gold standard for patient follow-up and treatment decisions [59, 60]. Our analysis reveals an average reduction in CMT of -127 ± 153 μm for patients with an initial BCVA score of < 69 letters and − 82.2 ± 82.1 μm for those with initial BCVA scores between 69 and 78 letters, consistent with results of Protocol T for the Management of DME observed for patients under treatment with IVR at one-year follow-up [61].

One of the main problems in DME treatment is the diverse factors that play a crucial role in its development and progression, making the treatment response variable among individuals. Patients with an initial score of < 69 letters and greater CMT have a significant opportunity for more remarkable visual improvement. Therefore, BCVA has been considered a prognostic and predictive biomarker [45]. According to findings from the DRCR.net Protocol T, patients with a baseline BCVA 20/50–20/320 experienced an average improvement of 18.3 letters with aflibercept, 13.3 letters with bevacizumab, and 16.1 letters with ranibizumab after 2 years. In contrast, patients with a baseline VA 20/32–20/40 showed average improvements of 7.8, 6.8, and 8.6 letters, respectively. The limited potential for functional improvement in patients with near-normal baseline vision likely reflects a ceiling effect, as these eyes are already approaching maximal visual function. According to an analysis of treatment patterns stratified by baseline BCVA, eyes with the best (> 20/25) and worst (< 20/200) starting vision receive the fewest anti-VEGF injections, and those with moderate vision (20/25–20/80) receive a significantly higher number of injections per year [62]. Our results indicated that patients with baseline BCVA < 69 letters and increased CMT required more frequent anti-VEGF injections, averaging 9.8 ± 1.6 in the first year (*p* < 0.001) to maintain disease stability. More frequent anti-VEGF injections during the first year of treatment are associated with a higher probability of maintaining long-term vision outcomes [63].

It has been hypothesized that, along with the increased frequency and number of injections, there is an added procedural risk: the elevation of IOP. It is well known that transient IOP elevations immediately after intravitreal injections are expected; however, the evidence of chronic IOP elevation related to repeated anti-VEGF injections is inconclusive [64–66]. Several studies have reported a positive correlation between a higher number of injections and a persistent elevation of IOP [64–69], as well as higher rates of elevated IOP in patients with pre-existing glaucoma [67], regardless of the anti-VEGF agent used. The proposed causes include the injection procedure itself, subclinical inflammation, recurrent episodes of transient post-injection elevations in IOP, decreased aqueous outflow due to the direct effect of anti-VEGF agents on nitric oxide levels, which decreases trabecular meshwork cell contractility, or indirect effect related to particle retention in ocular structures derived from the drug or syringes used for injection [68–73]. However, we found no statistically significant difference in IOP variation between these patients and those who required fewer treatments. These findings are consistent with data reported in previous large, randomized, multicenter clinical trials with up to three years of follow-up [74–76].

Another important aspect to emphasize is that in routine clinical practice, eyes with DME typically receive 2 to 4 anti-VEGF injections per year (in contrast to the 9 to 15 injections received in clinical studies) and experience poorer visual outcomes than patients in clinical trials [25, 62]. The significant treatment burden of diabetes has a major impact on patients’ lives, with factors affecting adherence. As a result, there are gaps in treatment, changes in anti-VEGF medications, and variations in treatment intervals in standard practice [62].

Despite the challenges, the rates of dropout, death, serious adverse events (including death), and systemic adverse events were similar in this study compared to what has been reported in other clinical trials [61, 63]. The most frequently reported adverse event was a hypertensive emergency, occurring in 7.7% of patients. In a previous trial by DRCR.net, which compared ranibizumab to laser photocoagulation for treating DME, there was no evidence of an increased cardiovascular risk associated with ranibizumab [77]. Additionally, an earlier trial involving anti-VEGF agents found that patients with wet age-related macular degeneration treated with bevacizumab experienced more significant systemic toxicity than those treated with ranibizumab [78]. However, a subsequent meta-analysis published by the Cochrane Collaboration did not confirm this finding [79, 80]. No increased risk of adverse events was consistently observed in our trial for patients. Additionally, there was no clinically significant increased risk of major ocular complications in patients treated with anti-VEGF drugs, such as glaucoma or endophthalmitis, or an increased risk of cataract progression and increased IOP.

The primary limitation of the study is the lack of information regarding each patient’s specific treatment (PRO-169 or IVR). The study remains blinded, as it is an ongoing clinical follow-up trial. Consequently, the researchers and the biostatistician conducting the analysis remain unaware of the intervention groups. Accordingly, all findings are presented based on the patient’s BCVA at the time of admission and the conclusion of their participation. Including patients with reasonable glycemic control and who do not require insulin treatment may narrow the sample of subjects. Furthermore, all the patients included had HbA1c levels below 9.5%. In Mexico, the prevalence of diabetes was 15.7% of the population in 2020, yet only 39% of individuals with diabetes achieved reasonable glycemic control [81]. Therefore, the findings reported in this study may appear conservative, considering the sample characteristics. Finally, the study mainly involves Mexican or Latino patients (with Mexican ancestry), most of whom live in Mexico. Factors such as socioeconomic status, healthcare access, and health issues like diabetes or hypertension which are common in the Mexican population, can affect outcomes. Therefore, it’s unclear whether the results apply to other populations with different characteristics.

## Conclusion

The results of this interim analysis offer preliminary insights into the potential benefits of a novel anti-VEGF agent. As the clinical trial is still ongoing and remains blinded, treatment allocation cannot be determined with certainty at this stage. Nevertheless, the interim findings support the efficacy of intravitreal anti-VEGF therapy, particularly among patients with lower baseline visual acuity (< 69 letters). Upon completion of the study, a final analysis will be required to confirm the efficacy and safety of PRO-169. The translational relevance of our research provides compelling evidence for ophthalmologists treating Latino patients with DME. The introduction of a new anti-VEGF agent could reduce treatment costs and increase therapy accessibility, while maintaining efficacy in the management of DME, ultimately contributing to improved patient care in this population.

## Supplementary Information

Below is the link to the electronic supplementary material.

Supplementary Material 1

## Data Availability

In addition to the summary statistics, the supplementary material provides the proof of principle for each model, which was performed openly in the Open Science Framework (https://osf.io) and is available as DOI 10.17605/OSF.IO/M9KAH.

## Abbreviations

BCVA: Best corrected visual acuity
CMT: Central macular thickness
DME: Diabetic macular edema
DR: Diabetic retinopathy
ETDRS: Early treatment diabetic retinopathy study
HbA1c: Glycated hemoglobin
IOP: Intraocular pressure
IVR: Intravitreal ranibizumab
VEGF: Vascular endothelial growth factor
