# Supplementary material for: One-year results of visual response following intravitreal novel anti-VEGF injection for diabetic macular edema in a Latino population
Source: Int J Retina Vitreous. 2025 Aug 1;11:89. doi: 10.1186/s40942-025-00719-9 (PMC12315349; doi:10.1186/s40942-025-00719-9)
Supplement: Supplementary file 1 — Supplementary Material 1 [file 40942_2025_719_MOESM1_ESM.docx]

One-year results of visual response following intravitreal novel anti-VEGF injection for diabetic macular edema in a Latino Population.

### Guillermo Salcedo-Villanueva^1, †^, Gisela Garcia-Sánchez^2, †^, Claudia Palacio-Pastrana^3^, Gerardo Gascón-Guzmán^4^, Aureliano Moreno-Andrade^5^, Oscar Olvera-Montaño^2^, Patricia Muñoz-Villegas^2,^ *

### Affiliations

^1^Asociación para Evitar la Ceguera en México, I.A.P., Mexico City, Mexico.

^2^Regional Medical Affairs Department, Laboratorios Sophia, Zapopan, Jalisco, Mexico.

^3^SalaUno Salud, S.A.P.I. de C.V., Mexico City, Mexico.

^4^Consultorio de Medicina Especializada, Nuevo Laredo, Tamaulipas, Mexico.,

^5^Retina Center, Tijuana, Baja California, Mexico.

^†^ These Authors contributed equally to this work and shared first authorship.

*Correspondence: Patricia Muñoz-Villegas, Regional Medical Affairs Department, Laboratorios Sophia, SA de CV., Paseo del Norte 5255, Guadalajara Technology Park, 45010, Zapopan, Jalisco, México. patricia.munoz@sophia.com.mx

-----------------------------------------------------------------------------------------------------

## **Table S1.** Selection Criteria

| **Inclusion Criteria** |
| --- |
| Age ≥ 18 years. |
| Diagnosis of diabetes mellitus (type 1 or 2) evidenced by using insulin or oral hypoglycemic medications or diagnosis for diabetes mellitus according to WHO and ADA criteria. |
| Can render informed consent. |
| HbA1c <9.5% in screening visits. |
| All men and women capable of reproduction may agree to use a barrier birth control method during the study and for three months after the last intravitreal injection is applied. |
| Only one eye may be randomized per participating individual; if both are eligible, the investigator may choose either eye according to their criteria. |
| BVCA, according to the Early Treatment Diabetic Retinopathy Study (ETDRS), was between < 78 (20/32 or worse) and > 24 (20/320 or better) within 8 days before the randomization. |
| Clinically evident diabetic macular edema, with central macular thickening. |
| Diabetic macular edema was demonstrated in an OCT scan (macular central thickness [measured in the 1-mm diameter central subfield] > 300μm for men and > 290μm for women) within 8 days before the randomization. |
| We present characteristics that enable an adequate fundus examination (e.g., transparent media, adequate pupil dilation, etc.). |
| **Exclusion Criteria** |
| Chronic kidney disease in renal failure (glomerular filtration rate <15 mL/min/1.73m^2^) requiring dialysis or transplantation, according to the Clinical Practice Guideline for the Management of Diabetes in Chronic Kidney Disease 2020 of the KDIGO. |
| Individuals with conditions that may compromise their participation during the study (unstable concomitant diseases, possible change of residence, etc.). |
| Individuals with poor glycemic control who have started insulin treatment within four months before the study. |
| Participation in another clinical study (at least 90 days must have elapsed between the finalization of their involvement in a previous essay and randomization in the present study). |
| Known allergies to the treatment. |
| Poorly controlled blood pressure (average of 3 readings while sitting with ≥160 mmHg systolic or ≥100 mmHg diastolic in the selection visit. |
| Heart attack or other cardiovascular event (cerebral vascular disease, transitory ischemia, hospitalization for cardiac insufficiency) during the four months before the start of the study, or patients with active myocardial insufficiency. |
| Previous systemic treatment with VEGF-related medications within four months before the start of the study. |
| Women of childbearing age who are pregnant, lactating, or planning to get pregnant within the period of the study. |
| Known allergy to anesthetic medications used during the procedures, intravitreal injection, and photocoagulation. |
| Diagnosis of non-diabetic macular edema. |
| Ophthalmic conditions interfere with the evaluation of BCVA (for example, foveal atrophy, pigmentary abnormalities, dense foveal exudates, etc.) |
| Additional conditions to DM may compromise the evaluation of the edema (for example, venous occlusions, uveitis, other inflammatory diseases, neovascular glaucoma, etc.) |
| Lens opacities that exceed one or more of the following, according to the LOCS III classification system: > NO3C3, > C2, > P1. |
| Previous history of anti-VEGF treatment for diabetic macular edema or any treatment for diabetic macular edema within four months before the study (corticosteroids, photocoagulation, etc.). |
| Anticipation of the need for pan-photocoagulation (for example, proliferative diabetic retinopathy or any other indication) during the study period or history of pan-photocoagulation within the 4 months before the start of the study. |
| History of ocular surgery (cataract extraction, intraocular surgery, aphakia, etc.) within 4 months prior to the start of the study or planned to occur within the study's time span. |
| Intraocular pressure > 21 mmHg, measured through Goldmann tonometry during the selection visit. |
| The presence of macular ischemia or an essential loss of perifoveal capillaries (avascular foveal zone more significant than 350μm) was demonstrated through fluorescein angiography during the selection visit. |
| Evidence of macular traction and hyaloid thickening in the OCT scan. |
| History of YAG capsulotomy within two months before the randomization. |
| Evidence of external ocular infections or any critical disease of the ocular surface. |
| History of vitrectomy. |

## **Table S2.** Patients who discontinued the clinical trial within 36 weeks or less of follow-up versus those who remained in the study.

| Population | | Safety population | Study population | p-value [95% CI] |
| --- | --- | --- | --- | --- |
| Number of patients (eyes) | | 75 | 278 | … |
| Sex, M / F (%) | | 42 (56) / 33 (44) | 145 (52.2) / 133 (47.8) | 0.554^b^ |
| Age, years ± SD | | 62.6 ± 8.8 | 62.1 ± 7.4 | 0.626, [-2.45, 1-48]^a^ |
| Glycosylated hemoglobin, g/dL % | | 7.4 ± 0.9 | 7.1 ± 0.9 | 0.067, [-0.43, 0.01]^a^ |
| Diagnosis of DM, years ± SD | | 15.3 ± 8.4 | 16.7 ± 8.4 | 0.188, [-0.71, 3.59]^a^ |
| Diagnosis of DME, years ± SD | | 0.8 ± 1.0 | 1.2 ± 1.6* | 0.048, [0.003, 0.77]^a^ |
| Central macular thickness, µm ± SD | | 475 ± 150** | 430 ± 114 | 0.005, [-76.55, -13.91]^a^ |
| BCVA-ETDRS letters ± SD | | 54.1 ± 13.9 | 58.5 ± 13.5* | 0.013, [0.94, 7.89]^a^ |
| BCVA-LogMAR ± SD | | 0.61 ± 0.27 | 0.53 ± 0.27* | 0.015, [-0.16, 0.02]^a^ |
| Intraocular pressure, mmHg ± SD | | 14.2 ± 2.4 | 14.5 ± 2.1 | 0.260, [-0.24, 0.88]^a^ |
| Reasons for Discontinuation of Participation, n (%) | AE | 18 (24) | … | … |
|  | Death | 1 (1.3) | … |  |
|  | Loss to follow-up | 29 (38.7) | … |  |
|  | Major protocol deviations | 19 (25.3) | … |  |
|  | Withdrawal of IC | 8 (10.7) | … |  |
| Ocular AE | | 7 (38.9) | 51 (30) | … |
| Systemic AE | | 11 (61.1) | 119 (70) | … |
| Serious AE | | 13 (72.2) | 23 (13.5) | … |
| Notes: ^a^t-test. Chi-square test, ^b^Chi-square test. For time after diagnosis of DM and BCVA, the study population is greater than the safety population. For CMT, the safety population is greater than the study population. *p<0.050, **p<0.010.  Abbreviations: AE, adverse events; BCVA, best corrected visual acuity; CI, confidence interval; DM, diabetes mellitus; DME, diabetic macular edema; IOP, intraocular pressure; F, female; M, male; SD, standard deviation. | | | | |

## **
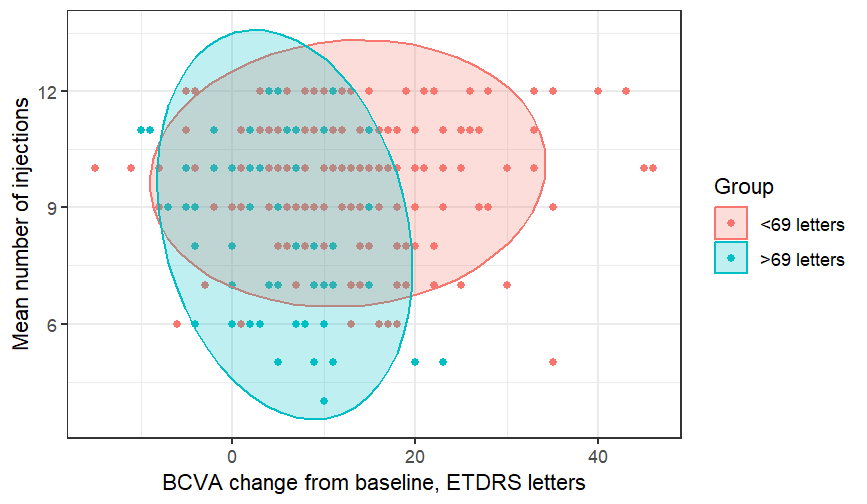
**

**Figure S1** displays a scatter plot with error ellipses, demonstrating the potential variations in the adjusted x- and y-coordinates at a 95 percent confidence level. The plot explores the relationship between changes in visual acuity and the average number of injections received by patients with an initial best corrected visual acuity (BCVA) letter score of < 69 than those with an initial BCVA of 69 to 78.


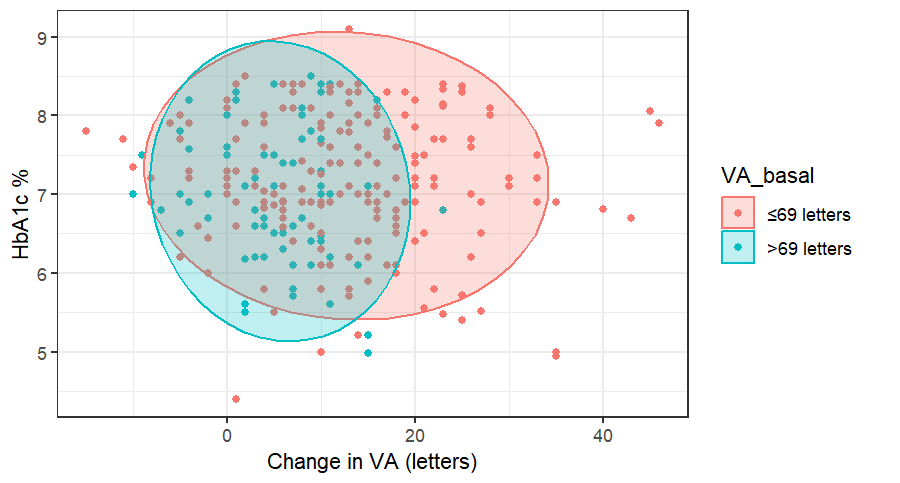


**Figure S2.** The scatter plot, which includes error ellipses, illustrates the potential variations in the adjusted x- and y-coordinates at a 95 percent confidence level. It depicts the relationship between changes in visual acuity and glycosylated hemoglobin levels after one year of intravitreal injections. This comparison is made between patients with an initial visual acuity of < 69 and those with an initial BCVA of 69 to 78.


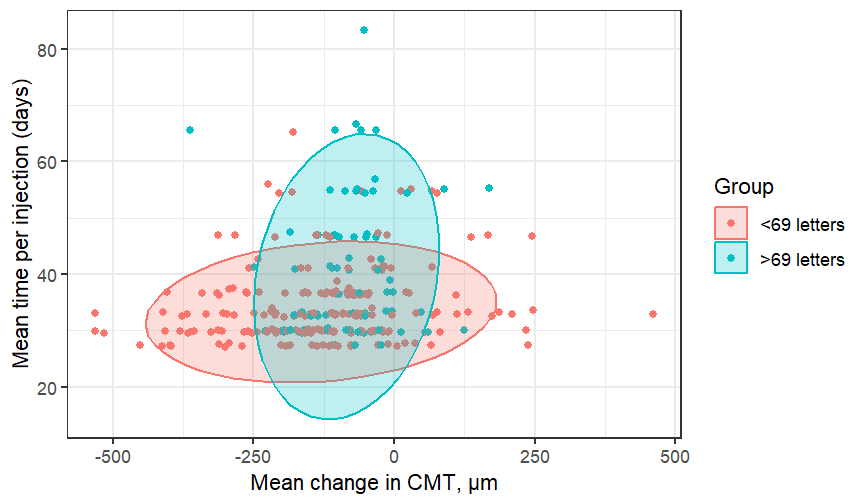


**Figure S3.** The scatter plot, which includes error ellipses, demonstrates the potential variations in the adjusted x- and y-coordinates at a 95 percent confidence level. It illustrates the change in central macular thickness after one year of intravitreal injections. It compares patients with an initial best corrected visual acuity (BCVA) letter score of < 69 to those with an initial BCVA of 69 to 78.

**
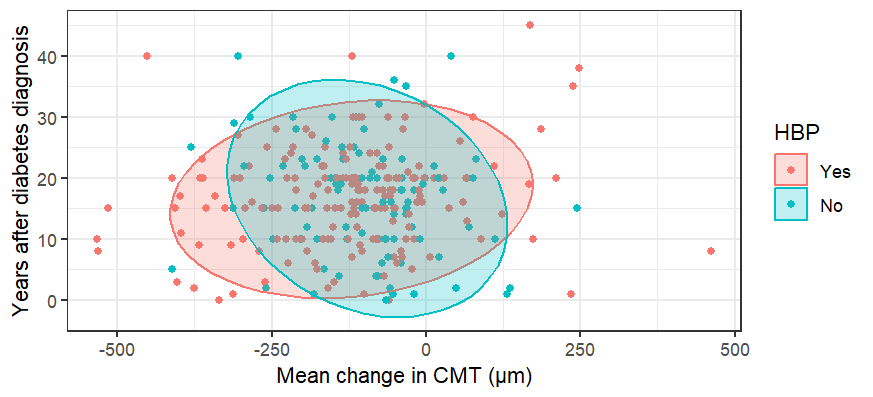
**

**Figure S4.** The scatter plot, which includes error ellipses, shows the potential variances of the adjusted x- and y-coordinates at a 95% confidence level. It illustrates the relationship between changes in central macular thickness and the number of years since a diabetes mellitus diagnosis for patients with and without systemic hypertension (high blood pressure).

**
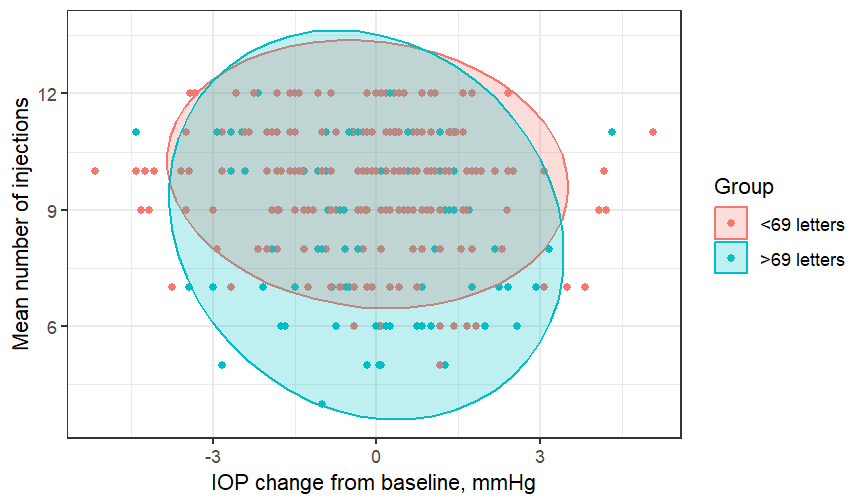
**

## **Figure S5.** The scatter plot, accompanied by error ellipses, illustrates the potential variances in the adjusted x- and y-coordinates at a 95 percent confidence level. It highlights the relationship between changes in intraocular pressure (IOP) and the average number of injections for patients with an initial best corrected visual acuity (BCVA) letter score of less than 69, compared to those with an initial BCVA between 69 and 78.

**
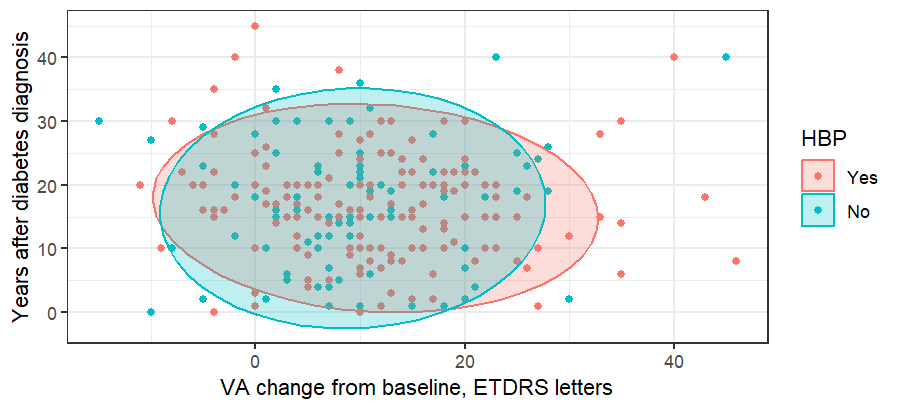
**

**Figure S6.** The scatter plot, which includes error ellipses, depicts the potential variations in the adjusted x- and y-coordinates at a 95 percent confidence level. It shows the relationship between changes in visual acuity and the number of years since diabetes diagnosis for patients with and without systemic hypertension (HBP, high blood pressure).
